# The Potential of *Thymus serpyllum* Essential Oil as an Antibacterial Agent against *Pseudomonas aeruginosa* in the Preservation of Sous Vide Red Deer Meat

**DOI:** 10.3390/foods13193107

**Published:** 2024-09-28

**Authors:** Miroslava Kačániová, Stefania Garzoli, Anis Ben Hsouna, Alessandro Bianchi, Maciej Ireneusz Kluz, Joel Horacio Elizondo-Luevano, Zhaojun Ban, Rania Ben Saad, Wissem Mnif, Peter Haščík

**Affiliations:** 1 Institute of Horticulture, Faculty of Horticulture and Landscape Engineering, Slovak University of Agriculture, Trieda Andreja Hlinku 2, 949 76 Nitra, Slovakia; 2 School of Medical & Health Sciences, University of Economics and Human Sciences in Warsaw, Okopowa 59, 01043 Warszawa, Poland; m.kluz@vizja.pl; 3 Department of Chemistry and Technologies of Drug, Sapienza University, P. le Aldo Moro, 5, 00185 Rome, Italy; stefania.garzoli@uniroma1.it; 4 Laboratory of Biotechnology and Plant Improvement, Centre of Biotechnology of Sfax, B.P “1177”, Sfax 3018, Tunisia; benhsounanis@gmail.com (A.B.H.); raniabensaad@gmail.com (R.B.S.); 5 Department of Environmental Sciences and Nutrition, Higher Institute of Applied Sciences and Technology of Mahdia, University of Monastir, Monastir 5000, Tunisia; 6 Department of Agriculture, Food and Environment, University of Pisa, Via del Borghetto 80, 56124 Pisa, Italy; alessandro.bianchi@phd.unipi.it; 7 Faculty of Agronomy, Universidad Autónoma de Nuevo León, Av. Francisco Villa S/N, Col. Ex Hacienda el Canadá, General Escobedo 66050, Nuevo León, Mexico; joel.elizondolv@uanl.edu.mx; 8 Zhejiang Provincial Key Laboratory of Chemical and Biological Processing Technology of Farm Products, Zhejiang Provincial Collaborative Innovation Center of Agricultural Biological Resources Biochemical Manufacturing, School of Biological and Chemical Engineering, Zhejiang University of Science and Technology, Hangzhou 310023, China; banzhaojun@zust.edu.cn; 9 Department of Chemistry, College of Sciences of Bisha, University of Bisha, P.O. Box 199, Bisha 61922, Saudi Arabia; wmoneef@ub.edu.sa; 10 Institute of Food Technology, Faculty of Biotechnology and Food Sciences, Slovak University of Agriculture, Trieda Andreja Hlinku 2, 949 76 Nitra, Slovakia; peter.hascik@uniag.sk

**Keywords:** antimicrobial agent, antibiofilm activity, wild thyme essential oil, sous vide deer meat, *Pseudomonas aeruginosa*

## Abstract

Foodborne infections caused by microbes are a serious health risk. Regarding this, customer preferences for “ready-to-eat” or minimally processed (MP) deer meat are one of the main risk factors. Given the health dangers associated with food, essential oil (EO) is a practical substitute used to decrease pathogenic germs and extend the shelf-life of MP meals. Nonetheless, further data regarding EO use in MP meals are required. In order to evaluate new, safer alternatives to chemicals for disease control and food preservation, this research was carried out in the following areas to assess the antibacterial and antibiofilm characteristics of *Thymus serpyllum* (TSEO) essential oil, which is extracted from dried flowering stalks. Furthermore, this study applied an essential oil of wild thyme and inoculated the sous vide deer meat with *Pseudomonas aeruginosa* for seven days at 4 °C in an effort to prolong its shelf-life. Against *P. aeruginosa*, the essential oil exhibited potent antibacterial action. The findings of the minimal biofilm inhibition concentration (MBIC) crystal violet test demonstrated the substantial antibiofilm activity of the TSEO. The TSEO modified the protein profiles of bacteria on glass and plastic surfaces, according to data from MALDI-TOF MS analysis. Moreover, it was discovered that *P. aeruginosa* was positively affected by the antibacterial properties of TSEO. The anti-*Pseudomonas* activity of the TSEO was marginally higher in vacuum-packed sous vide red deer meat samples than in control samples. The most frequently isolated species from sous vide deer meat, if we do not consider the applied bacteria *Pseudomonas aeruginosa*, were *P. fragi, P. lundensis*, and *P. taetrolens*. These results highlight the antibacterial and antibiofilm qualities of TSEO, demonstrating its potential for food preservation and extending the shelf-life of deer meat.

## 1. Introduction

Due to the presence of many genetically movable elements, innate and acquired resistance mechanisms, and bacterial genomes with multiple copies, the Gram-negative *Pseudomonas* genus comprises more than sixty species with significant metabolic adaptability and ecological variety [1]. Of the many species that make up this genus, *Pseudomonas aeruginosa* is an opportunistic pathogen that is relevant to medicine and veterinary care [2]. In healthy humans or animals, this pathogen is normally present in the gut microbiota [1], but it also causes nosocomial infections, lung infections in patients with cystic fibrosis, disseminated infections in immunocompromised people [2], and in livestock (e.g., causing mastitis in dairy cows) and companion animals [3]. *P. aeruginosa* is one of the most important antibiotic-resistant (AMR) “priority pathogens,” according to the World Health Organization, because of its remarkable capacity to evade the effects of antibiotics [4]. Circulating strains of *P. aeruginosa* are now resistant to several classes of antibiotics due to their ubiquity, their ability to successfully exploit a variety of environmental niches, and the acquisition of antibiotic resistance as a result of selective pressure exerted by sub-inhibitory concentrations of antibiotics [5]. *Pseudomonas aeruginosa* is a crucial species for the formation of biofilms and a model bacteria for studying biofilms. Autogenous extracellular polymeric substances (EPS) constitute the main structural component of biofilms, which function as a scaffold to enclose bacteria on surfaces, protect them from environmental stresses, and prevent phagocytosis [6]. The characteristics of bacteria in biofilms were different from those of planktonic development. In particular, bacteria in biofilms are considerably less vulnerable to host defenses, disinfectants, and antibiotics [7]. *P. aeruginosa* forms biofilms by attaching itself to surfaces that are conducive to development, such as food and medical equipment. This is followed by the production of microcolonies, which is the last stage of the process, and maturation, which involves the expression of matrix polymers [8].

Given its low fat and high protein composition, deer meat is a valuable diet from a nutritional perspective [9]. Consumers are becoming more interested in game meat as they look for a local, balanced, and healthy diet that also considers sustainability and ethical issues [10]. Meat hygiene is hampered by the difficulty of standardizing hunting and environmental circumstances. The issue is made worse by variations in the methods used for hunting and handling game meat, which affect its microbial load (ML), as well as the absence of data reporting [11]. The plethora of suggested interventions that follow the soiling of game corpses with intestinal contents as a result of an incorrect shot or during evisceration is an illustration of these various hunting and handling techniques. According to several studies [11,12], game killed with an exact shot in the thoracic region had lower bacterial counts than game killed with a shot in the abdominal region. It has been hypothesized that increased bacterial contamination, particularly with pathogens, is the result of apparent contamination of the meat with intestinal contents [13].

In recent times, there has been an increasing interest in sous vide mild heat treatment among researchers, households, and the catering industry. This is because it offers a practical way to increase meat yields, enhance sensory qualities like tenderness and juiciness, as well as improve oxidative stability, microbiological quality, and shelf-life of meat products [14,15].

The traditional sous vide method uses a single cooking temperature of between 55 and 70 °C for variable durations, depending on the type, thickness, and amount of connective tissue in the meat [16]. On the other hand, as storage duration can have a big impact on the microbiological characteristics of cooked meat, it is necessary to investigate the storage stability of meat products made using this method. Moreover, germs on the meat’s surface have the ability to grow and cause spoiling and off-flavors [17,18].

The food business has long utilized artificial preservatives, with antimicrobial preservatives being the most common. However, recent research suggests that consuming chemical additives can cause allergies, intoxications, cancer, and other degenerative disorders [19]. Customers depreciate them as a result, which makes it necessary to hunt for different options. In place of conventional antimicrobial drugs, this quest has produced novel agents with a natural origin [20]. Essential oils (EOs) can prolong the shelf-life of beef by reducing the amount of chemical deterioration that occurs during the storage and marketing phases, as well as by getting rid of undesirable microbes.

For this reason, it was decided to use the plant essential oil *Thymus serpyllum* in in vitro conditions in this work, as well as its use in *Pseudomonas aeruginosa* bacteria inoculated on deer meat. Compared to Gram-negative bacteria, Gram-positive bacteria seem to be a little more susceptible to the effects of EO [21]. Indeed, wild thyme has demonstrated potential antibacterial effect in vitro against food pathogens such as *Salmonella*, *Staphylococcus*, *E. coli*, *Klebsiella*, *Pseudomonas*, and *Enterococcus*, at concentrations ranging from 5 to 20 µL EO. Thymol and carvacrol are also efficient against bacteria such as *P. aeruginosa* and *S. aureus* [22].

These substances work together to weaken the plasma membrane, change the pH, and throw off the inorganic ion balance, all of which inhibit the growth of these microorganisms. According to Lambert et al. [23], the spice that was most efficient against the most germs was wild thyme oil. They explored the effects of essential oils derived from different plants on lactic acid bacteria, *S. typhimurium*, *E. coli*, and *L. monocytogenes*, as well as their bactericidal and bacteriostatic properties. As shown by Lambert et al. [23], thymol, carvacrol, and other lipophilic chemicals found in wild thyme oil, like eugenol and limonene, interact with bacterial membranes to alter their structure and improve their permeability.

To the best of our knowledge, this is the first study on the application of *Thymus serpyllum* essential oil to the preservation of meat, particularly sous vide red deer meat. TSEO is a naturally occurring preservative that has the power to regulate microorganisms. The TSEO and its main active ingredients, carvacrol and thymol, show promise and value as antiseptics in the food industry due to their aromaticity, antibacterial and antifungal properties, and safety. The flavor of deer meat can be enhanced with the use of TSEO. Additionally, by giving the meat more sweetness and tenderness, it can enhance how palatable deer meat is. Nevertheless, a thorough investigation into the TSEO’s effects on sous vide deer meat after *Pseudomonas aeruginosa* inoculation has never been conducted. The aim of this study was to investigate the antibacterial characteristics and efficacy of TSEO in inhibiting *P. aeruginosa* biofilm formation in vitro. In order to increase the shelf-life of the sous vide red deer meat, this study also looked into the survival of *P. aeruginosa* inoculated onto meat that was cooked using the sous vide cook-chill method and then kept at 4 °C for 7 days.

## 2. Materials and Methods

### 2.1. Characteristics of Essential Oil

The wild thyme (*Thymus serpyllum*) essential oil (TSEO) utilized in this study was obtained from Hanus s.r.o. in Nitra, Slovakia. Dried flowering stalks were steam-distilled to extract them. During the analyses, it was kept at 4 °C in the dark.

### 2.2. Chemical Composition of TSEO

The chemical composition of TSEO was previously published [24]. The main components of TSEO were determined by gas chromatography/mass spectrometry (GC/MS) and gas chromatography (GC-FID), with thymol accounting for 18.8%, carvacrol for 17.4%, o-cymene for 15.4%, and geraniol for 10.7%. Other components such as γ-terpinene (8.1%), linalool (5.3%), geranyl acetate (4.4%), and borneol (2.3%) were detected with lower percentage mean values [24].

### 2.3. Antimicrobial Activity

#### 2.3.1. Pseudomonas Strain Preparation

The aim of this study was to examine the antibacterial and antibiofilm properties of *Pseudomonas aeruginosa*. Additionally, the effects of sous vide cooking on deer meat were examined, and it was shown that the cooking method extended shelf-life by lowering microbial numbers and antimicrobial activity. The *Pseudomonas aeruginosa* CCM 1959 strain used was obtained from a microbial collection, the Czech Collection of Microorganisms (Brno, Czech Republic). Mueller–Hinton agar (MHA, Oxoid, Basingstoke, UK) was used to cultivate the bacteria, and it was incubated for 24 h at 37 °C. Following the adjustment of the bacterial culture’s optical density to the 0.5 McFarland standard, which corresponds to 1.5 × 10^8^ CFU/mL, 100 µL of the inoculum was tested for antibacterial and antibiofilm activity before being introduced to deer meat samples. After being inoculated with *P. aeruginosa*, the deer meat samples were carefully mixed for three minutes at room temperature to ensure uniform dissemination of the infection.

#### 2.3.2. Disc Diffusion Method

Disk diffusion was the method used to evaluate the antibacterial efficacy of the TSEO. The Mueller–Hinton Broth (MHB, Oxoid, Basingstoke, UK) medium was used to cultivate bacterial cultures for 24 h at 37 °C. After cultivation, distilled water was used to bring the bacterial density down to the 0.5 McFarland standard, or 1.5 × 10^8^ CFU/mL. Then, on Mueller–Hinton Agar (MHA, Oxoid, Basingstoke, UK), 100 μL of the bacterial solution was equally distributed. Adherent to the agar plates were sterile 6 mm disks that had been soaked with 10 μL of TSEO. Three separate measurements of the inhibition zones surrounding each disk were taken following a 24 h incubation period at 37 °C.

#### 2.3.3. Minimal Inhibition Concentration

The bacteria were allowed to incubate in Mueller-Hinton broth (MHB, Oxoid, Basingstoke, UK) at 37 °C for a duration of 24 h. The cultures were applied in 150 μL quantities to each well of a 96-well microplate after being adjusted to an optical density corresponding to the 0.5 McFarland standard. To attain ultimate concentrations varying from 10 mg/mL to 0.00488 mg/mL, 150 μL quantities of TSEO were additionally introduced. After that, the microplate was incubated at 37 °C for 24 h. MHB with the TSEO served as the negative control, while MHB with a bacterial inoculum served as the positive control. After incubation, a Glomax spectrophotometer (Promega Inc., Madison, WI, USA) was used to detect absorbance at 570 nm. The lowest EO concentration that inhibits 50% of bacterial growth is known as the MIC_50_, and the concentration that inhibits 90% of growth is known as the MIC_90_. The experiment was carried out in triplicate to guarantee precision and dependability.

### 2.4. Antibiofilm Activity

#### 2.4.1. Crystal Violet Assay

A thorough investigation of the Minimal Biofilm Inhibitory Concentration (MBIC) was carried out [25]. Throughout the day, bacterial suspensions were grown in Mueller–Hinton broth (MHB, Oxoid, Basingstoke, UK) at 37 °C in an aerobic environment. Following the incubation period, an inoculum was created to reach the 0.5 McFarland standard optical density. 100 μL of the bacteria and 100 μL of the TSEO were added to each well of a 96-well microtiter plate. 100 μL of the TSEO was added to the first column, and then, using a pipette, the concentration was diluted twice to reach 0.00488 mg/mL and 10 mg/mL. MHB with bacterial inoculum was used to maintain maximum growth control, while MHB with TSEO was used as the negative control. Following a 24 h incubation period at 37 °C, the supernatant was disposed of, and the wells were thoroughly cleaned three times using 250 μL of saline solution. Finally, they were allowed to dry for half an hour at room temperature. After that, the wells were stained for 15 min with 200 μL of 0.1% *w*/*v* crystal violet. This was followed by multiple washings with deionized water and drying. After solubilizing the samples with 200 μL of 33% acetic acid, a Glomax spectrophotometer (Promega Inc., Madison, WI, USA) was used to detect absorbance at 570 nm. The concentration at which the absorbance was either equal to or lower than the negative control was identified as the MBIC. The doses that block 50% and 90% of biofilm growth, respectively, were designated as MBIC_50_ and MBIC_90_.

#### 2.4.2. Antibiofilm Detection by MALDI-TOF MS Biotyper

The protein breakdown during biofilm formation was evaluated using the Bruker Daltonics MALDI-TOF MicroFlex device (Brucker, Bremen, Germany). Initially, 50 mL polypropylene tubes holding tiny glass and plastic were filled with 100 μL of *P. aeruginosa* bacterial inoculum and 20 mL of MHB. While control tubes were left untreated, experimental tubes were treated with TSEO until a final concentration of 0.1% was reached. Tubes were shaken at 170× *g* for seven days at 37 °C. Using sterile cotton swabs, biofilms from glass and plastic surfaces were collected every day and then transferred to target plates. Additionally examined were planktonic cells from untreated control samples. The control bacterial cultures were centrifuged for one minute at 12,000× *g* after 300 µL of culture material was added. After three ultrapure water washes, pellets were centrifuged one more and placed on target plates for examination. Plates with a 10 mg/mL α-cyano-4-hydroxycinnamic acid matrix were coated with reconstituted pellets and swabs (1 μL each). Plates were dried and then calibrated mass-to-charge ratios between 2000 and 20,000 using linear positive mode MALDI-TOF analysis. Automated techniques were used to assess eighteen typical global spectra (MSPs), in order to determine Euclidean distances and create dendrograms [25,26].

### 2.5. Shelf-Life Extension of Sous Vide Deer Meat

#### Deer Meat Preparation

The deer (*Cervus elaphus*) meat samples from the *biceps femoris muscle* of a 5-year-old deer that came from shooting grounds in Slovakia were the subject of this investigation. The meat evaluated in this study had 72.06 g of water, 0.73 g of fat, 22.04 g of protein, and 0.031 g of cholesterol per 100 g. Four kilograms of thigh flesh in all was gathered and initially kept refrigerated before being sent to a microbiological facility for additional examination. The meat was then divided into 483 separate samples by slicing it into 5 g chunks with a sterile knife. Three raw deer meat samples were distributed on day 0, and 240 samples each were distributed on days 1 and 7 for the control and treated groups. These samples were distributed over different time points. Groups for control and treatment were assigned to each 5 g chunk of deer meat. The meat was combined with a 1% (*v*/*w*) solution of TSEO dissolved in sunflower oil for the treatment group. After that, a Concept vacuum packer from Chocen, Czech Republic, was used to vacuum pack each sample. After being mixed with the TSEO solution, the treatment groups were vacuum-packed, and the control samples were placed in polyethylene bags.

Each sample received 100 µL of *P. aeruginosa* and the TSEO solution during the preparation phase. The quick mixing phase, which lasted for about a minute before vacuum sealing, was performed with great care to avoid contamination. Throughout our trial, we experimented with many ways to cook fresh deer meat, including

1. After being kept in polyethylene bags at 4 °C, fresh deer meat was cooked for 5 to 25 min at temperatures between 50 and 65 °C.
2. Control vacuum: Deer meat was cooked in a water bath for 5 to 25 min at temperatures between 50 and 65 °C after being vacuum-sealed in polyethylene bags at 4 °C.
3. The deer meat was treated with essential oil (1% TSEO solution), vacuum-packed, and stored at 4 °C. It was then cooked in a water bath at 50 to 65 °C for 5 to 25 min.
4. Contamination with *P. aeruginosa*: Deer meat that was injected with the bacteria, vacuum-packed, and kept at 4 °C until exposed was cooked in a water bath for 5 to 25 min at temperatures between 50 and 65 °C.
5. Treatment with *P. aeruginosa* and essential oil: Deer meat was treated with both *Pseudomonas aeruginosa* and a 1% TSEO solution. It was then vacuum-packed, kept at 4 °C, and cooked in a water bath for 5 to 25 min at temperatures between 50 and 65 °C.

As controls, samples of raw deer meat were prepared on day zero. The TSEO or *P. aeruginosa* was added to these samples, which were then left to rest for a full day before being cooked sous vide in a CASO SV1000 device from Arnsberg, Germany. The meat was placed within high-barrier polyethylene bags, which are renowned for their sturdiness, moisture resistance, and capacity to tolerate temperatures between −30 °C and +100 °C. Because these bags are specifically made without microplastics or plasticizers like bisphenol A, food safety during extended refrigeration is guaranteed.

### 2.6. Microbiological Evaluation of Deer Meat

Periodic microbiological assessments were carried out during the experiment, as reported by Kačániová et al. [27]. The samples were heat treated after being stored for 24 h at 4 °C, and they were subsequently evaluated on a prearranged basis. First, a 1:10 dilution ratio was achieved by placing 5 g of red deer meat samples in sterile stomacher bags and diluting them with 45 mL of peptone water. For thirty minutes, the samples were homogenized with the stomacher device. Following homogenization, standard plate count agar medium was covered with 100 µL aliquots from the appropriate dilutions, and the incubation period was 30 min with shaking. Violet Red Bile Lactose Agar (VRBL; Oxoid, Basingstoke, UK) was used to cultivate coliform bacteria, and it was incubated at 37 °C for 24 to 48 h. For the Total Viable Count (TVC), Plate Count Agar (PCA; Oxoid, Basingstoke, UK) was used, and it was incubated at 30 °C for 48 to 72 h. Based on discernible development in these media, viable counts were established. By cultivating on Pseudomonas agar (PA; Oxoid, Basingstoke, UK) with CN supplement and incubating at 35 °C for 24 to 48 h, *P. aeruginosa* were found.

### 2.7. Bacteriota Identification by MALDI-TOF MS Biotyper

Using well-established reference libraries, the MALDI-TOF MS Biotyper system from Bruker Daltonics in Bremen, Germany, was used to identify microorganisms generated from samples of deer thigh tissue. An initial stock was made up of 50% acetonitrile, 47.5% water, and 2.5% trifluoroacetic acid in order to prepare the matrix solution. 500 µL of pure acetonitrile, 475 µL of filtered water, and 25 µL of 10% trifluoroacetic acid were combined to create this stock solution. Then, according to earlier instructions [28], the “HCCA matrix solution” was made in a 250 µL Eppendorf flask, completely combined with the organic solvent, and purchased from Aloqence Science in Vrable, Slovakia. Then, eight different colonies from the Petri dishes underwent the appropriate processing. The biological material from these colonies was put into an Eppendorf flask, combined with 300 µL of distilled water, and centrifuged using a ROTOFIX 32A centrifuge from ITES in Vranov, Slovakia, at 10,000× *g* for two minutes. After centrifugation, 900 µL of ethanol was added, and the pellet was allowed to air dry at room temperature (20 °C) until the supernatant was removed. Lastly, the pellet was mixed with 30 µL of 70% formic acid and 30 µL of acetonitrile. The MALDI-TOF analysis yielded scores that were interpreted according to specific criteria. Specifically, scores in the range of 2.000 to 2.299 suggested genus identification with potential species identification, scores between 1.700 and 1.999 indicated likely genus identification, and scores below 1.700 were deemed unreliable.

### 2.8. Statistical Analyses

Every assessment was conducted in triplicate, and the results are presented as mean values ± standard deviation (SD). A one-way ANOVA (CoStat version 6.451, CoHort Software, Pacific Grove, CA, USA) and Duncan’s multiple range test (MRT) were used for the statistical analysis, with a significance level of *p* < 0.05 for sample differentiation.

Software from SAS Institute, Cary, NC, USA called JMP Pro 17.0 was used to create the graphical depiction.

## 3. Results

### 3.1. Antimicrobial Activity

The findings of the disc diffusion method and minimal inhibitory concentration (MIC) used to assess the antibacterial activity of the TSEO are shown in Table 1. A 17.33 mm zone of inhibition against *P. aeruginosa* demonstrated the strong antibacterial activity of the TSEO. The antibiotic ciprofloxacin, on the other hand, had greater effectiveness with a zone of inhibition that measured 33.33 mm. The MIC values were ascertained to be 0.134 mg/mL for MIC_50_ and 0.146 mg/mL for MIC_90_.

### 3.2. Antibiofilm Activity

Additionally, the crystal violet biofilm against *P. aeruginosa* experiment was used to determine the minimal biofilm inhibition concentration, which was determined to be MBIC_50_ at 0.227 mg/mL and MBIC_90_ at 0.236 mg/mL (Table 1).

**Figure 1:**
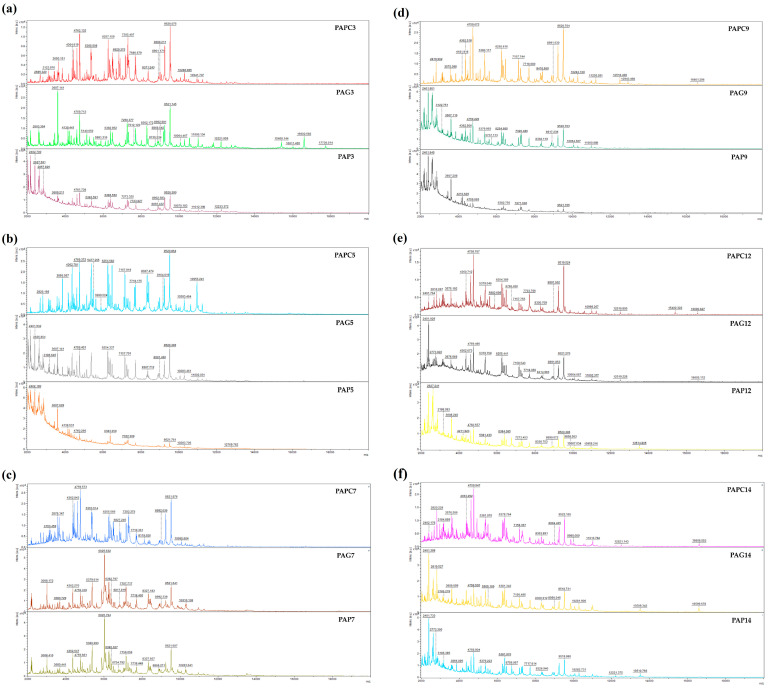
Representative MALDI-TOF mass spectra of *P. aeruginosa*: (**a**) 3rd day; (**b**) 5th day; (**c**) 7th day; (**d**) 9th day; (**e**) 12th day; (**f**) 14th day. PA = *P. aeruginosa*; G = glass; P = plastic; and PC = planktonic cells.

The MS spectra of *P. aeruginosa* at various developmental stages treated with TSEO on glass and plastic surfaces are shown in Figure 1a–f. The spectra of planktonic cells that were employed as controls are also provided. There was some fluctuation in the protein spectra numbers between the experimental and control groups on the third treatment day (SEPC 3, SEG 3, and SES 3). On the other hand, spectrum evolution similarities indicated that both groups were producing related proteins. Significant variations in the mass spectrum evolution were seen by the fifth day (SEPC 5, SEG 5, and SES 5), indicating the influence of TSEO on biofilm stability. By the ninth day, notable differences had been apparent, especially in the biofilms’ spectra on glass and plastic, which suggested that the TSEO had been efficient in disrupting the biofilms. However, until the end of the experiment, certain commonalities in the evolution of the spectrum remained. These results show that the TSEO can disturb the homeostasis of *P. aeruginosa* biofilms; significant effects were seen on both surfaces from day 3 to day 9. This implies that longer-term effective suppression of biofilm growth may be possible at greater TSEO concentrations.

**Figure 2:**
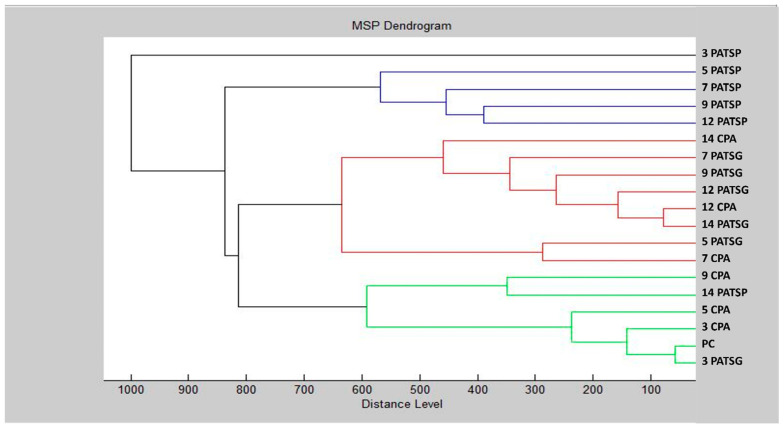
A dendrogram of *P. aeruginosa* generated using MSPs of the planktonic cells and the control. PA = *P. aeruginosa*; C = glass; P = plastic; and PC = planktonic cells.

The control groups on days 12 and 14 on the glass surface showed the lowest MSP distances on day 3, as well as during the early phases of biofilm formation on the planktonic cells, according to the dendrogram in Figure 2. The plastic surface had a larger MSP distance than the glass surface, indicating that the TSEO had a more pronounced inhibitory impact on *P. aeruginosa* biofilms on the plastic surface. However, the largest increase in MSP distance was observed on plastic surfaces between days 3 and 5 of the experiment. An additional investigated component was the minimal spectral peak (MSP) distances between planktonic cells and controls. Over the course of this study, the MSP distances of the experimental group increased. Specifically, on the third day of the trial employing the glass surface, the MSP distance of the experimental group was the lowest. For the experimental group, the maximum length of the MSP distance was reached by days 3 and 5, especially on the plastic surface. Comparable patterns were noted for days 7, 9, and 12. The results of this investigation demonstrate how the TSEO inhibits and negatively impacts *P. aeruginosa* biofilm formation on glass and plastic surfaces.

### 3.3. Microbiological Quality of Sous Vide Deer Meat Samples

#### 3.3.1. Number of Microorganisms

**Figure 3:**
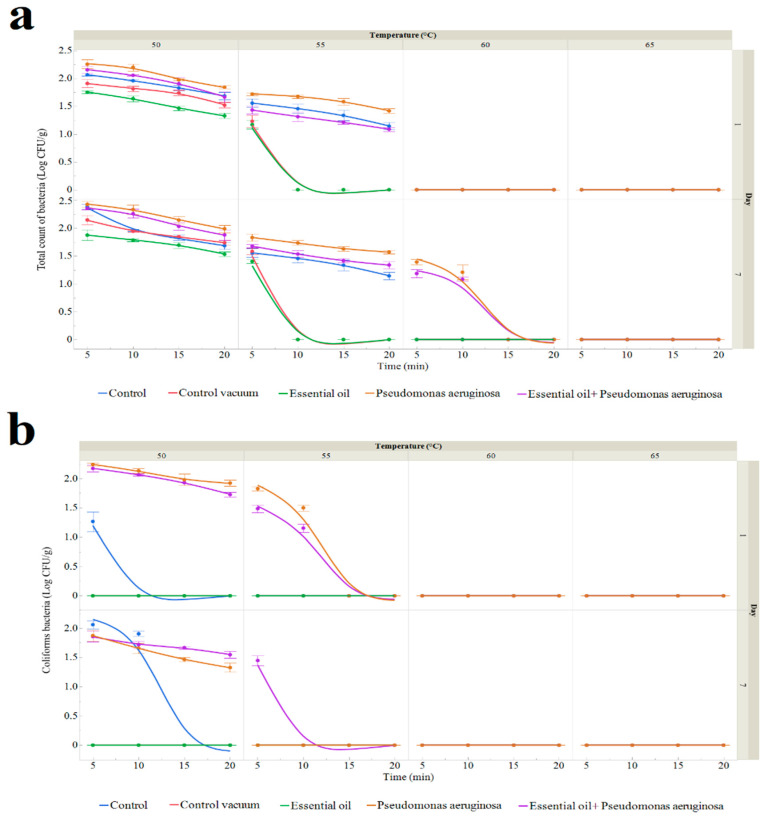

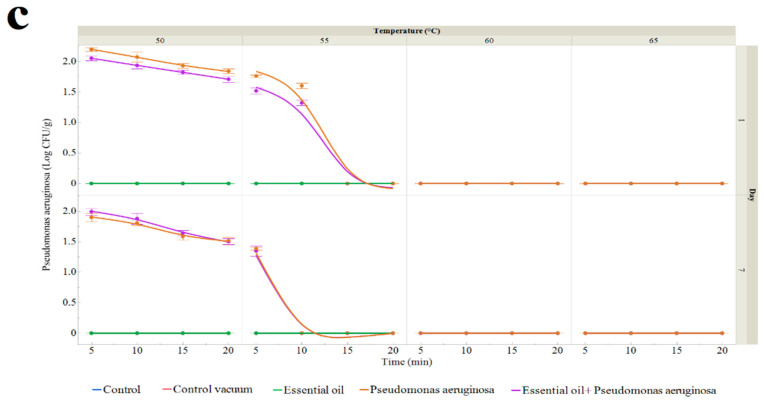
The results of the bacteria count (represented in log CFU/g) on the first and seventh day, treated for periods of 5 to 20 min at 50 to 65 °C: *Pseudomonas aeruginosa*, (**c**) Coliform bacteria (**b**), and total count of bacteria (**a**). The three samples’ means (±SD) constitute the data. Control: after being vacuum-packed in polyethylene bags and stored at 4 °C, fresh sample was treated for 5 to 25 min at 50 to 65 °C. Control vacuum: after being vacuum-packed in polyethylene bags and stored at 4 °C, fresh sample was treated for 5 to 25 min at 50 to 65 °C. Essential oil: a newly vacuum-packed sample treated with 1% EO was stored at 4 °C and allowed to sit at 50–65 °C for 5 to 20 min. *Pseudomonas aeruginosa*: *P. aeruginosa* was applied to a vacuum-packed fresh sample that was stored at 4 °C and treated for 5 to 20 min at 50 to 65 °C. EO + *Pseudomonas aeruginosa*: *Pseudomonas aeruginosa* and 1% EO were added to a vacuum-packed fresh sample, which was stored at 4 °C and treated for 5 to 20 min at 50 to 65 °C.

The total viable count (TVC) of sous vide red deer meat samples that were exposed to various temperatures, periods, the TSEO, and *P. aeruginosa* treatments is shown in Figure 3a and Table S1. The control samples were red deer meat that was raw, undercooked, and unpackaged. Day 0 initial assessments revealed no coliform bacteria present and a TVC of 2.56 ± 0.11 log CFU/g. Day 1 TVC in the sous vide red deer meat control group ranged from 1.14 ± 0.07 log CFU/g (55 °C for 20 min) to 2.07 ± 0.02 log CFU/g (50 °C for 5 min). Compared to samples in the control group without vacuum packing, those that were vacuum-packed displayed lower TVC (Table S1). In particular, samples that were vacuum-packed ranged from 1.23 ± 0.11 log CFU/g (55 °C for 5 min) to 1.91 ± 0.07 log CFU/g (50 °C for 5 min); samples that were treated with TSEO ranged from 1.17 ± 0.08 log CFU/g (55 °C for 5 min) to 1.75 ± 0.02 log CFU/g (50 °C for 5 min); samples that were treated with *P. aeruginosa* ranged from 1.42 ± 0.04 log CFU/g (55 °C for 20 min) to 2.26 ± 0.08 log CFU/g (50 °C for 5 min); and samples that were treated with TSEO and inoculated with *P. aeruginosa* ranged from 1.09 ± 0.04 log CFU/g (55 °C for 20 min) to 2.16 ± 0.06 log CFU/g (50 °C for 5 min). By day 7, the TVC in the control group ranged from 1.14 ± 0.07 log CFU/g (55 °C for 20 min) to 2.38 ± 0.05 log CFU/g (50 °C for 5 min). Vacuum-packaged sous vide deer meat ranged from 1.58 ± 0.06 log CFU/g (55 °C for 5 min), and the TSEO-treated samples ranged from 1.40 ± 0.03 log CFU/g (55 °C for 5 min) to 1.88 ± 0.10 log CFU/g (50 °C for 5 min) (Figure 3a, Table S1). For samples with *P. aeruginosa* application, the TVC ranged from 1.21 ± 0.13 log CFU/g (60 °C for 10 min) to 2.43 ± 0.05 log CFU/g (50 °C for 5 min), and those treated with the TSEO and inoculated with *P. aeruginosa* ranged from 1.08 ± 0.04 log CFU/g (60 °C for 10 min) to 2.43 log CFU/g (50 °C for 5 min).

Figure 3a and Table S2 display the quantity of coliform bacteria (CB) in samples of sous vide red deer meat. Day 0 has zero CB counts. Only the first treatment of temperature and time resulted in the detection of CB at 1.27 ± 0.17 log CFU/g in the control group, which was packaged in polyethylene bags under aerobic conditions.

On day 7, the CB counts in the control group varied between 1.90 ± 0.04 log CFU/g for the group that received 10 min of treatment at 50 °C and 2.06 ± 0.07 log CFU/g for the group that received 5 min of treatment at 50 °C (Figure 3b and Table S2). There were no CBs in the group where the vacuum packaging and TSEO were implemented. Day 7 CB ranged from 1.33 ± 0.08 log CFU/g at 50 °C for 20 min to 1.87 ± 0.10 log CFU/g at 50 °C for 5 min in the group that received *P. aeruginosa* application. The CB counts varied between 1.45 ± 0.08 log CFU/g at 55 °C for 5 min and 1.86 ± 0.09 log CFU/g in the group that received both the *P. aeruginosa* application and TSEO treatment.

*P. aeruginosa* numbers were only found in the final two groups during the storage period, as shown in Figure 3c and Table S3. The group that received the *P. aeruginosa* inoculation showed a range of counts on day 1 of 1.32 ± 0.05 log CFU/g (55 °C for 10 min) to 2.20 ± 0.03 log CFU/g (50 °C for 5 min). On the other hand, the group that was given TSEO treatment and inoculated with P. aeruginosa exhibited counts that varied between 1.32 ± 0.05 log CFU/g (at 55 °C for 10 min) and 2.05 ± 0.04 log CFU/g (at 50 °C for 5 min). By the seventh day, counts in the *P. aeruginosa* group varied from 1.39 ± 0.03 log CFU/g (55 °C for 5 min) to 1.90 log CFU/g (50 °C for 5 min). In contrast, counts in the group that had TSEO treatment and received the *P. aeruginosa* inoculation varied from 1.35 ± 0.09 log CFU/g (50 °C for 5 min) to 1.99 log CFU/g (50 °C for 5 min).

#### 3.3.2. Identification of Microorganisms of Sous Vide Deer Meat

**Figure 4:**
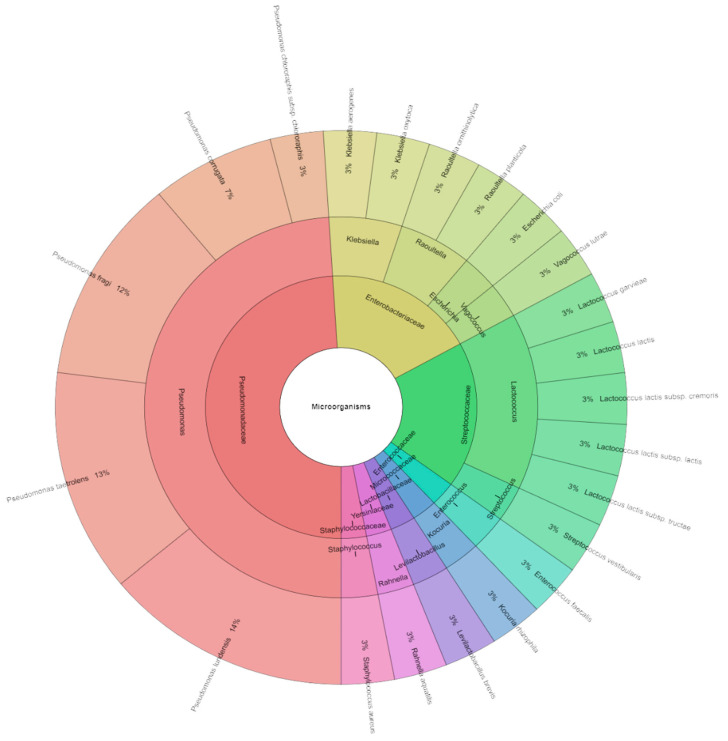
Krona chart: Bacteriota from deer sous vide meat on first day in control groups.

**Figure 5:**
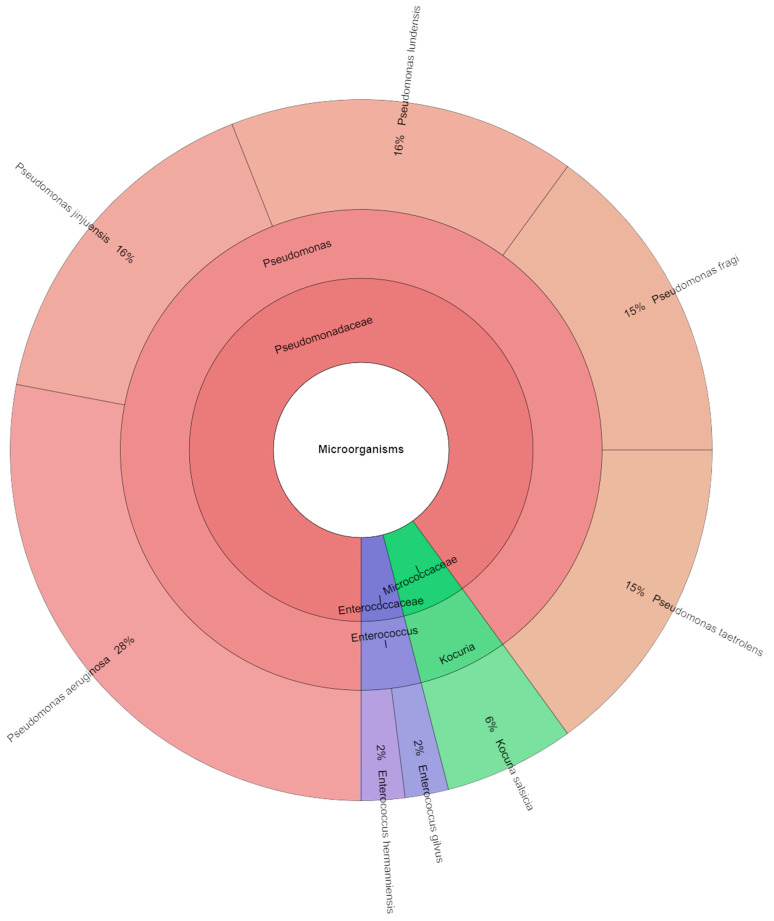
Krona chart: Bacteriota from deer sous vide meat on first day in experimental groups.

**Figure 6:**
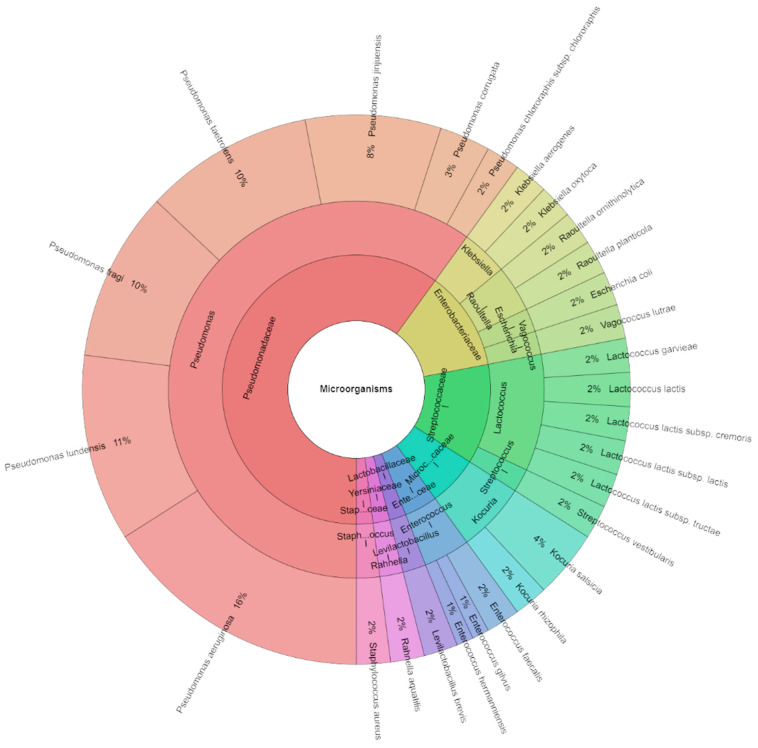
Krona chart: Bacteriota from deer sous vide meat on first day in all groups together.

In this study, we also focused on the bacterial profile of sous vide deer meat in the control and experimental groups individually and together on the first day and the seventh day of storage. Species, genera, and families isolated from red deer sous vide meat samples on the first storage day are shown in Figure 4. Using mass spectrometry, 145 isolates were found, with scores as high as two in the control group. These isolates came from 22 species, 12 genera, and 7 families. The Pseudomonadaceae, Streptococcaceae, and Enterobacteriaceae families contained the most commonly detected species. First day sous vide deer meat samples were used to isolate *Pseudomonas ludensis* (14%), which was followed by *Pseudomonas taetrolens* (13%), and *Pseudomonas fragi* (12%). Species, genera, and families that were isolated from experimental groups are displayed in Figure 5. Mass spectrometry was used to identify 125 isolates in total, with scores as high as 2 in the experimental group. Eight species, three genera, and three families comprised these isolates. The Pseudomonadaceae family contained the majority of the species that were identified. The most common species recovered from the sous vide deer meat samples in the experimental groups on day one was *P. aeruginosa* (28%), which was inoculated on the samples, followed by *P. lundensis* and *P. jinjuensis*, which combined accounted for 16% of the samples. Figure 6 was pushed together in all groups. On the first day, 315 isolates were isolated in total. On the day of storage, a total of 27 species of bacteria belonging to 12 genera and 8 families were identified in all stories. The most isolated species on the first day of storage was *P. aeruginosa* (16%), which was inoculated on meat, followed by *P. fragi*, *P. lundensis*, and *P. taetrolens*.

**Figure 7:**
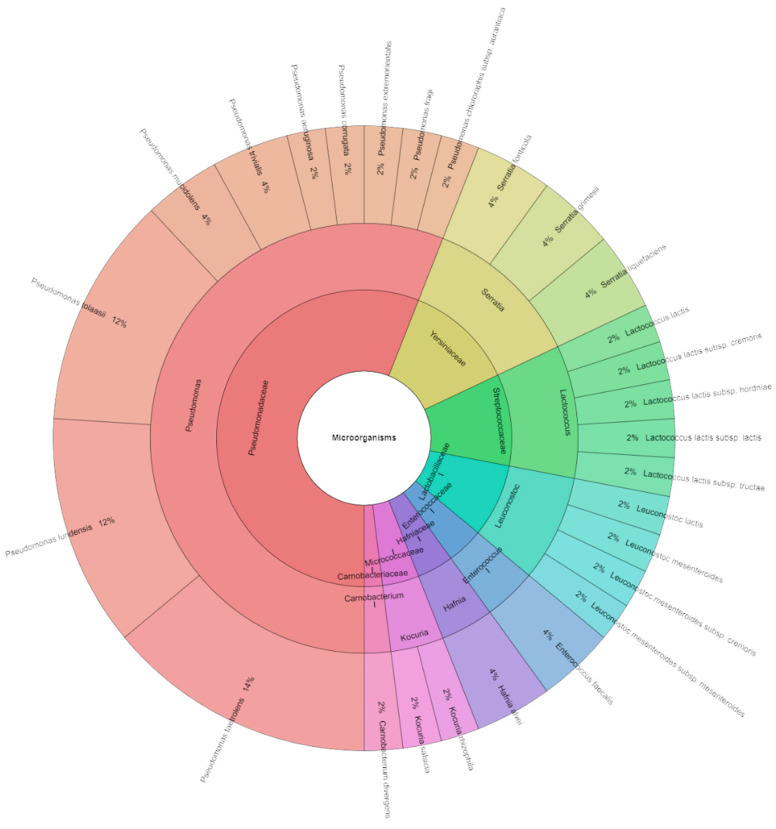
Krona chart: Bacteriota from deer sous vide meat on seventh day in control groups.

**Figure 8:**
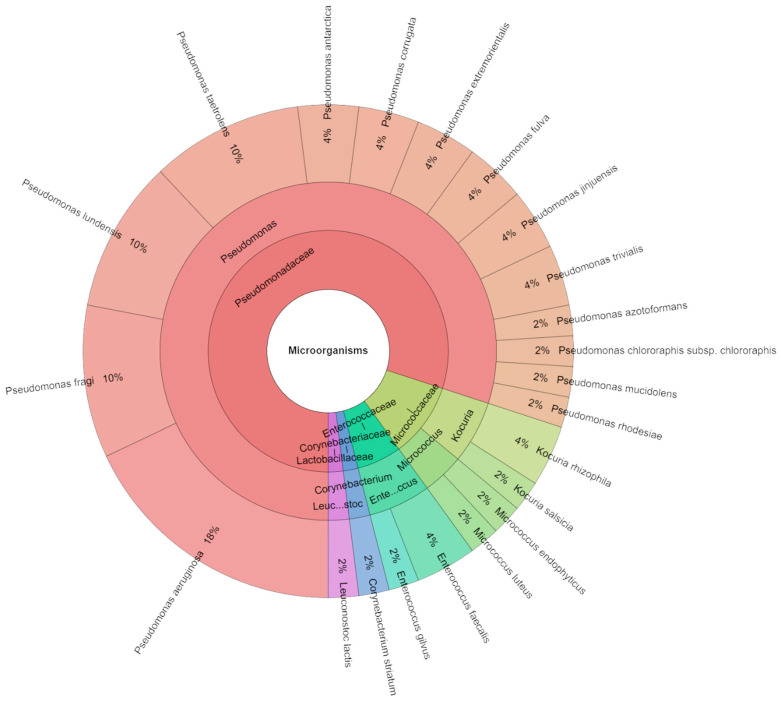
Krona chart: Bacteriota from deer sous vide meat on seventh day in experimental groups.

**Figure 9:**
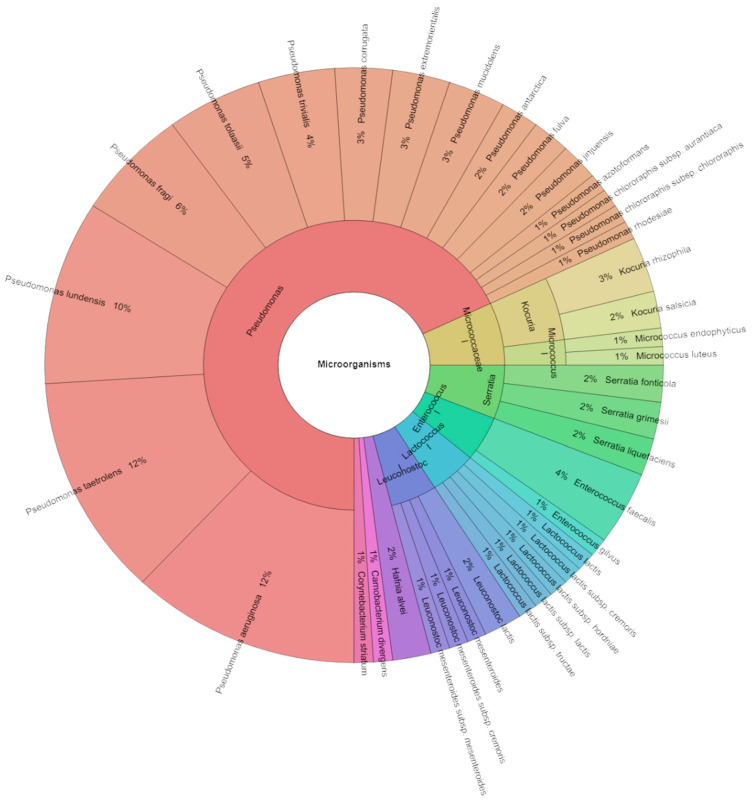
Krona chart: Bacteriota from deer sous vide meat on seventh day in all groups together.

Figure 7 displays the species, genera, and families that were separated from red deer sous vide meat samples on the seven storage days. Mass spectrometry was used to identify 285 isolates, with a control group scoring as high as two. These isolates were from 8 genera, 8 families, and 27 species. The most often discovered species belonged to the groups Pseudomonadaceae, Streptococcaceae, and Enterobacteriaceae. *P. taetrolens* (14%) was the most isolated from seven day sous vide deer meat samples. Figure 8 shows the species, genera, and families that were separated from the experimental groups. A total of 255 isolates were identified by mass spectrometry, with scores as high as 2 in the experimental group. These isolates were divided into 22 species, 6 genera, and 5 families. Most of the discovered species were members of the Pseudomonadaceae family. *P. aeruginosa*, which was inoculated on the samples, was the most often recovered species from the sous vide deer meat samples (14%) in the experimental groups on day seven. It was followed by *P. lundensis* and *P. tolasii*, which together accounted for 12% of the samples. In every group, Figure 9 was pushed together. A total of 490 isolates were identified on the seventh day. In all tales on the day of storage, a total of 38 species of bacteria from 10 genera and 10 families were found. Following *P. fragi*, *P. lundensis*, and *P. taetrolens* as the most isolated species on the first day of storage was *P. aeruginosa* (12%), which was inoculated on meat.

## 4. Discussion

Researchers are looking for novel antimicrobials due to the ongoing resistance of harmful bacteria to antibiotics. This finding could bolster the notion that EOs’ and their components’ antibacterial potentials have been thoroughly studied. Due to their intricate chemical makeup, EOs present a viable treatment option for the issue of antibiotic resistance. Their antibacterial activity and mechanism of action have already been covered in a number of articles [29,30]. The chemical composition of the essential oils of *Thymus serpyllum* has been previously established [24]. The main components were thymol (18.8%), carvacrol (17.4%), o-cymene (15.4%), and geraniol (10.7%). Components such as γ-terpinene (8.1%), linalool (5.3%), geranyl acetate (4.4%), borneol (2.3%), and others were less representative. In another study, 52 compounds in *T. serpyllum* EO were identified, accounting for 99.37% of the composition. The main compounds were thymol (21.5%) and carvacrol (18.7%) [31]. The primary antimicrobial components of thymes were found to be monoterpene phenols (thymol and carvacrol), monoterpene hydrocarbons (terpinene), monoterpene alcohols (borneol), and bicyclic sesquiterpene hydrocarbons (caryophyllene) in several studies on the chemical compositions of the essential oils [32,33].

Thyme essential oils are well-known for their abundance of bioactive substances, including terpenes, flavonoids, and phenolic acids, as well as their variety of chemotypes [34,35,36,37]. Their biological content determines their qualities, according to earlier research [38]. Using the disc diffusion method and MIC determination, this work examined the antibacterial properties of wild thyme essential oil against *P. aeruginosa*. According to a different study, the TSEO and its constituents had substantial antibacterial action against both Gram-positive and Gram-negative bacteria as well as other microorganisms. TSEO showed only mediocre antibacterial efficacy against *C. tropicalis*, *S. aureus*, and *Y. enterocolitica* with the disc diffusion method. Inhibitory activity against *B. subtilis*, *E. faecalis*, *C. albicans*, *C. krusei*, and *C. glabrata* was found to be moderate. Strong inhibitory activity was demonstrated by *P. aeruginosa* [24]. The minimal inhibition concentration was used in the studies by Nikolić et al. [39] to test the antimicrobial activity against the bacteria *Streptococcus mutans*, *Streptococcus salivarius*, *Streptococcus sanguinis*, *Streptococcus pyogenes*, *Enterococcus faecalis*, *Pseudomonas aeruginosa*, *Lactobacillus acidophilus*, and *Staphylococcus aureus*. The TSEO showed the strongest activity against all tested microorganisms. A significant percentage of the phenolic component thymol is present in the oil, and a correlation has been shown between the antibacterial activity attained by a particular EO and its chemical composition, suggesting that this substance may be directly responsible for the action. A further justification for this assumption could be the fact that prior research [40,41] has shown thymol to be a potent antibacterial agent. *Haemopylus influenzae* and *H. parainfluenzae* were the strains most susceptible to the thyme EO treatment; the MIC value of thyme EO ranged from 0.156 to 0.187 mg/mL, which reduced their development. Higher MIC values, 1.5–1.75 mg/mL, were found in the *P. aeruginosa* case [42]. These results with *P. aeruginosa* showed higher antimicrobial activity than the previous study. Numerous investigations have examined the antibacterial efficacy of distinct *Thymus* chemotypes against a range of pathogens, such as *P. aeruginosa* and *Haemophilus* species [43,44,45]. The two chemotypes that have demonstrated the best antibacterial activity against these bacteria are *T. vulgaris* ct. thymol and *T. vulgaris* ct. carvacrol. In comparison to *P. aeruginosa*, geraniol and trans-thujan-4-ol/terpinen-4-ol chemotypes were less effective [46]. These findings corroborate earlier research showing the thymol chemotype thyme essential oil’s effectiveness against the examined microorganisms.

The antibiofilm activity of TSEO was evaluated with the crystal violet method and MALDI-TOF MS Biotyper. The most effective treatment against all of the tested microorganisms utilized in different investigations was TEO distilled from plant material that was taken at the start of the flowering phase. When tested against *P. aeruginosa*, this EO had the greatest inhibitory rate (72.93%) [42].

These results showed a very good potential for TSEO as an antibiofilm agent. In vitro microbiological tests showed that TEO caused a significant inhibition of biofilm biomass of both *S*. Typhimurium and *B. cereus* food isolates compared to untreated controls, as reported in the study of Sateriale et al. [47]. The treated and untreated samples altered MALDI-TOF mass spectra clearly demonstrate the variations in protein synthesis. Numerous studies have suggested that aberrations in protein synthesis are connected to the development of biofilms and their breakdown with the addition of EO. Furthermore, Božik et al. [48] used MALDI-TOF MS to evaluate the differences in protein production of bacteria under EO stress. The findings demonstrated that EOs affect the synthesis of proteins associated with stress, membranes, and biofilms in addition to ribosomal proteins. The antibiofilm activity of TSEO was assessed against *Bacillus subtilis* and *Stenotrophomonas maltophilia* in a separate investigation. TSEO was discovered to have outstanding antibacterial and anti-biofilm qualities on a range of surfaces utilizing the MALDI-TOF MS Biotyper. It came out that the MALDI-TOF MS Biotyper was a useful method for determining the various stages of biofilm formation [24].

The antimicrobial activity of TSEO against *P. aeruginosa* on the sous vide deer meat samples was evaluated in this study. Results show a good antimicrobial effect of TSEO against all microorganisms evaluated during 7 days of storage. It is essential to utilize safe and efficient preservation techniques that stop the deterioration of meat products and help stop the development of outbreaks of foodborne illness. In addition to high pressure and ionizing radiation, some of the current methods for preserving meat and meat products include heating, chilling, and packaging [49]. Furthermore, meat products can be kept from spoiling due to pathogenic bacteria and foodborne pathogens by using chemical preservatives and additives such as nitrites and nitrates [50]. However, the growing usage of artificial food additives has brought up a number of hazardous and carcinogenic issues as well as being linked to allergic reactions [51,52]. Because artificial ingredients are thought to be damaging to health, customers increasingly demand that food be free of them, including chemically generated food preservatives and antimicrobials. Several investigations have shown that essential oils used as preservatives have antibacterial qualities in vitro [34,53]. Recent studies, for example, have shown that thyme essential oil has antibacterial action against a variety of foodborne pathogens [54,55,56]. This suggests that thyme essential oil may be used to limit the spread of pathogenic bacteria and increase the shelf-life of food products [57]. The effectiveness of adding essential oils to food is being investigated in a number of studies [34,58], and there has been a recent surge in research on the use of EOs to inhibit the formation of biofilm on food and environmental surfaces [59,60,61].

The most often found organisms in sous vide deer meat belonged to the families Pseudomonadaceae, Streptococcaceae, and Enterobacteriaceae. *Pseudomonas ludensis* was the most isolated species from the Pseudomonadace family in the control and experimental groups. *Pseudomonas taetrolens* and *Pseudomonas fragi* were the next most isolated species. Furthermore, representatives of the genera *Escherichia*, *Enterobacter*, *Klebsiela,* and *Lactococcus* were identified from the meat. Similar results of sous vide deer meat were found in different studies, where, with a frequency of 6% each, *Pseudomonas fragi*, *Pseudomonas gessardii*, *Pseudomonas graminis*, *Pseudomonas libanensis*, and *Pseudomonas lundensis* were the most common species recovered. Additionally, at a frequency of 5% each, *Hafnia alvei* and *Bacillus cereus* were identified [28]. Many factors influence the microbiological quality of meat from hunted animals, the most prevalent being improper wound placement, gastrointestinal fluid or feces during expulsion contaminating muscle, and inadequate or delayed chilling [62]. Enterobacteriaceae, *Lactobacillus*, and *Pseudomonas* species are frequently identified in chilled meat. In rotten meat, *Pseudomonas* species predominate [63]. Fresh meat that has been kept in an aerobic environment has been found to deteriorate due to *Pseudomonas* bacteria. In aerobic circumstances and at low temperatures, they are among the quickest-developing organisms [64]. The first step in developing methods for preserving meat would be to identify the microbial communities present in the flesh. Another way to improve meat safety would be to assess the packing and storage conditions for the meat in addition to using natural antimicrobials. In this regard, antibacterial qualities found in many plant extracts, particularly essential oils, make them potentially useful for meat preservation [65].

## 5. Conclusions

According to this research, 1.0% wild thyme EO applied to deer meat along with vacuum packing effectively inhibits the growth of coliform bacteria, *Pseudomonas aeruginosa*, and the total viable count. When food microorganisms are rendered inactive, food safety is enhanced, and shelf-life is positively impacted. TSEO, a mildly flavored natural antibacterial, can be used to extend the freshness of vacuum-packed deer meat. Further investigation is necessary to further the suppression of total viable numbers. In conclusion, this study highlights the antibacterial and antibiofilm capabilities of the TSEO, both in vitro and in relation to the preservation of red deer meat in vacuum-packed packaging. These characteristics imply that it might be applied to food preservation to guarantee food safety and prevent food from going bad, especially when paired with other state-of-the-art processing and packaging methods. Using wild thyme essential oil can help prevent *Pseudomonas aeruginosa* contamination and extend the shelf-life of sous vide red deer meat while upholding quality and safety standards.

## Figures and Tables

**Table 1:** Antimicrobial and antibiofilm activity of the *Thymus serpyllum* essential oil (TSEO) against *Pseudomonas aeruginosa.* Data are presented as mean values ± standard deviation (SD) of three tests.

Inhibition Zone (mm)	TSEO	Ciprofloxacin
*Pseudomonas aeruginosa*	17.33 ± 0.58	33.33 ± 0.56
Minimal inhibition concentration (mg/mL)	MIC_50_	MIC_90_
*Pseudomonas aeruginosa*	0.134 ± 0.09	0.146 ± 0.05
Minimal biofilm inhibition concentration (mg/mL)	MBIC_50_	MBIC_90_
*Pseudomonas aeruginosa*	0.227 ± 0.07	0.236 ± 0.08

## Data Availability

The original contributions presented in this study are included in the article/Supplementary Materials, further inquiries can be directed to the corresponding author.

## Supplementary Materials

The following supporting information can be downloaded at: https://www.mdpi.com/article/10.3390/foods13193107/s1, Table S1: Total viable count (log CFU/g) of sous vide red deer meat samples after storage 1, and 7 days treated in a water bath at temperatures between 50 and 65 °C for 5 to 20 min. Data are the mean (bars indicate ± SD) of 3 red deer meat samples; Table S2: Total coliform bacteria (log CFU/g) of sous vide red deer meat samples after storage 1, and 7 days treated in a water bath at temperatures between 50 and 65 °C for 5 to 20 min. Data are the mean (bars indicate ± SD) of 3 red deer meat samples; Table S3: *P. aeruginosa* count (log CFU/g) of sous vide red deer meat samples after storage for 1 and 7 days treated in a water bath at temperatures between 50 and 65 °C for 5 to 20 min. Data are the mean (bars indicate ± SD) of 3 red deer meat samples.
