# Supplementary material for: The Potential of Thymus serpyllum Essential Oil as an Antibacterial Agent against Pseudomonas aeruginosa in the Preservation of Sous Vide Red Deer Meat
Source: Foods. 2024 Sep 28;13(19):3107. doi: 10.3390/foods13193107 (PMC11476099; doi:10.3390/foods13193107)
Supplement: Supplementary file 1 [file foods-13-03107-s001.zip › foods-3206925-supplementary.pdf]

Table S1. Total viable count (log CFU/g) of sous-vide deer meat samples after 1 and 7 days of storage, treated in a water bath at temperatures between 50 and 65 °C for 5 to 20 min. Control: deer meat samples placed in polyethylene bags without vacuum. Control vacuum: deer meat samples vacuum-packed in polyethylene bags. Essential oil: deer meat samples treated with 1% EO and vacuum-packed. *Pseudomonas aeruginosa*: deer meat samples inoculated with *Pseudomonas aeruginosa* and vacuum-packed. Essential oil + *Pseudomonas aeruginosa:* deer meat samples treated with 1% EO and inoculated with *Pseudomonas aeruginosa* and vacuum-packed.

Treatment	Temperature (°C)	Time (min)	Total count of bacteria (log CFU/g)	
			Days	
			1	7
Control	50	5	2.07±0.02^{b,A}	2.38±0.05^{a,A}
		10	1.96±0.02^{a,B}	1.96±0.02^{a,B}
		15	1.83±0.05^{a,C}	1.83±0.05^{a,C}
		20	1.69±0.07^{a,D}	1.69±0.07^{a,D}
	55	5	1.56±0.08^{a,DE}	1.56±0.08^{a,DE}
		10	1.46±0.08^{a,EF}	1.46±0.08^{a,EF}
		15	1.34±0.10^{a,F}	1.34±0.10^{a,F}
		20	1.14±0.07^{a,G}	1.14±0.07^{a,G}
	60	5	n.d.^{a,H}	n.d.^{a,H}
		10	n.d.^{a,H}	n.d.^{a,H}
		15	n.d.^{a,H}	n.d.^{a,H}
		20	n.d.^{a,H}	n.d.^{a,H}
	65	5	n.d.^{a,H}	n.d.^{a,H}
		10	n.d.^{a,H}	n.d.^{a,H}
		15	n.d.^{a,H}	n.d.^{a,H}
		20	n.d.^{a,H}	n.d.^{a,H}
Control vacuum	50	5	1.91±0.07^{b,A}	2.15±0.08^{a,A}
		10	1.81±0.05^{b,AB}	1.95±0.02^{a,B}
		15	1.74±0.05^{b,B}	1.85±0.01^{a,C}
		20	1.52±0.05^{b,C}	1.74±0.05^{a,D}
	55	5	1.23±0.11^{b,D}	1.58±0.06^{a,E}
		10	n.d.^{a,E}	n.d.^{a,F}
		15	n.d.^{a,E}	n.d.^{a,F}
		20	n.d.^{a,E}	n.d.^{a,F}
	60	5	n.d.^{a,E}	n.d.^{a,F}
		10	n.d.^{a,E}	n.d.^{a,F}
		15	n.d.^{a,E}	n.d.^{a,F}
		20	n.d.^{a,E}	n.d.^{a,F}
	65	5	n.d.^{a,E}	n.d.^{a,F}
		10	n.d.^{a,E}	n.d.^{a,F}
		15	n.d.^{a,E}	n.d.^{a,F}
		20	n.d.^{a,E}	n.d.^{a,F}
Essential oil	50	5	1.75±0.02^{a,A}	1.88±0.10^{a,A}
		10	1.64±0.05^{b,B}	1.79±0.03^{a,AB}
		15	1.46±0.04^{b,C}	1.70±0.06^{a,B}
		20	1.33±0.05^{b,D}	1.53±0.04^{a,C}
	55	5	1.17±0.08^{b,E}	1.40±0.03^{a,D}
		10	n.d.^{a,F}	n.d.^{a,E}
		15	n.d.^{a,F}	n.d.^{a,E}

		20	n.d.^{a,F}	n.d.^{a,E}
	60	5	n.d.^{a,F}	n.d.^{a,E}
		10	n.d.^{a,F}	n.d.^{a,E}
		15	n.d.^{a,F}	n.d.^{a,E}
		20	n.d.^{a,F}	n.d.^{a,E}
	65	5	n.d.^{a,F}	n.d.^{a,E}
		10	n.d.^{a,F}	n.d.^{a,E}
		15	n.d.^{a,F}	n.d.^{a,E}
		20	n.d.^{a,F}	n.d.^{a,E}
Pseudomonas aeruginosa	50	5	2.26±0.08^{b,A}	2.43±0.05^{a,A}
		10	2.20±0.06^{a,A}	2.34±0.08^{a,A}
		15	1.98±0.02^{b,B}	2.15±0.07^{a,B}
		20	1.84±0.02^{b,C}	1.99±0.07^{a,C}
	55	5	1.72±0.03^{b,D}	1.83±0.07^{a,D}
		10	1.68±0.03^{a,D}	1.74±0.05^{a,D}
		15	1.58±0.06^{a,E}	1.63±0.04^{a,E}
		20	1.42±0.04^{b,F}	1.57±0.04^{a,E}
	60	5	n.d.^{b,G}	1.39±0.05^{a,F}
		10	n.d.^{b,G}	1.21±0.13^{a,F}
		15	n.d.^{a,G}	n.d.^{a,G}
		20	n.d.^{a,G}	n.d.^{a,G}
	65	5	n.d.^{a,G}	n.d.^{a,G}
		10	n.d.^{a,G}	n.d.^{a,G}
		15	n.d.^{a,G}	n.d.^{a,G}
		20	n.d.^{a,G}	n.d.^{a,G}
Essential oil+ *Pseudomonas aeruginosa*	50	5	2.16±0.06^{b,A}	2.36±0.02^{a,A}
		10	2.06±0.01^{b,B}	2.26±0.08^{a,A}
		15	1.91±0.11^{a,C}	2.03±0.06^{a,B}
		20	1.67±0.09^{b,D}	1.88±0.10^{a,C}
	55	5	1.43±0.07^{b,E}	1.68±0.03^{a,D}
		10	1.31±0.08^{b,F}	1.54±0.06^{a,E}
		15	1.21±0.03^{b,G}	1.42±0.04^{a,F}
		20	1.09±0.04^{b,H}	1.34±0.06^{a,G}
	60	5	n.d.^{b,I}	1.19±0.08^{a,H}
		10	n.d.^{b,I}	1.08±0.04^{a,I}
		15	n.d.^{a,I}	n.d.^{a,L}
		20	n.d.^{a,I}	n.d.^{a,L}
	65	5	n.d.^{a,I}	n.d.^{a,L}
		10	n.d.^{a,I}	n.d.^{a,L}
		15	n.d.^{a,I}	n.d.^{a,L}
		20	n.d.^{a,I}	n.d.^{a,L}

Values are presented as mean ± standard deviation (SD) of of 3 deer meat samples. $^{a\text{-}b}$ Differents superscripts lowercase letters indicate statistical different value within row (Duncan's MRT, $p \leq 0.05$). $^{A\text{-}L}$ Differents superscripts uppercase letters indicate statistical different value within column for each treatment (Duncan's MRT, $p \leq 0.05$). n.d.= not detected (value = 0.00).

Table S2. Total coliforms bacteria (log CFU/g) of sous-vide deer meat samples after 1 and 7 days of storage, treated in a water bath at temperatures between 50 and 65 °C for 5 to 20 min. Control: deer meat samples placed in polyethylene bags without vacuum. Control vacuum: deer meat samples vacuum-packed in polyethylene bags. Essential oil: deer meat samples treated with 1% EO and vacuum-packed. *Pseudomonas aeruginosa*: deer meat samples inoculated with *Pseudomonas aeruginosa*and vacuum-packed. Essential oil + *Pseudomonas aeruginosa:* deer meat samples treated with 1% EO and inoculated with *Pseudomonas aeruginosa* and vacuum-packed.

**Treatment**	**Temperature (°C)**	**Time (min)**	**Coliforms bacteria (log CFU/g)**	
			**Days**	
			**1**	**7**
Control	50	5	$1.27\pm0.17^{b,A}$	$2.06\pm0.07^{a,A}$
		10	n.d.^{b,B}	$1.90\pm0.04^{a,B}$
		15	n.d.^{a,B}	n.d.^{a,C}
		20	n.d.^{a,B}	n.d.^{a,C}
	55	5	n.d.^{a,B}	n.d.^{a,C}
		10	n.d.^{a,B}	n.d.^{a,C}
		15	n.d.^{a,B}	n.d.^{a,C}
		20	n.d.^{a,B}	n.d.^{a,C}
	60	5	n.d.^{a,B}	n.d.^{a,C}
		10	n.d.^{a,B}	n.d.^{a,C}
		15	n.d.^{a,B}	n.d.^{a,C}
		20	n.d.^{a,B}	n.d.^{a,C}
	65	5	n.d.^{a,B}	n.d.^{a,C}
		10	n.d.^{a,B}	n.d.^{a,C}
		15	n.d.^{a,B}	n.d.^{a,C}
		20	n.d.^{a,B}	n.d.^{a,C}
Control vacuum	50	5	n.d.^{a,A}	n.d.^{a,A}
		10	n.d.^{a,A}	n.d.^{a,A}
		15	n.d.^{a,A}	n.d.^{a,A}
		20	n.d.^{a,A}	n.d.^{a,A}
	55	5	n.d.^{a,A}	n.d.^{a,A}
		10	n.d.^{a,A}	n.d.^{a,A}
		15	n.d.^{a,A}	n.d.^{a,A}
		20	n.d.^{a,A}	n.d.^{a,A}
	60	5	n.d.^{a,A}	n.d.^{a,A}
		10	n.d.^{a,A}	n.d.^{a,A}
		15	n.d.^{a,A}	n.d.^{a,A}
		20	n.d.^{a,A}	n.d.^{a,A}
	65	5	n.d.^{a,A}	n.d.^{a,A}
		10	n.d.^{a,A}	n.d.^{a,A}
		15	n.d.^{a,A}	n.d.^{a,A}
		20	n.d.^{a,A}	n.d.^{a,A}
Essential oil	50	5	n.d.^{a,A}	n.d.^{a,A}
		10	n.d.^{a,A}	n.d.^{a,A}
		15	n.d.^{a,A}	n.d.^{a,A}
		20	n.d.^{a,A}	n.d.^{a,A}
	55	5	n.d.^{a,A}	n.d.^{a,A}
		10	n.d.^{a,A}	n.d.^{a,A}
		15	n.d.^{a,A}	n.d.^{a,A}

		20	n.d.^{a,A}	n.d.^{a,A}
	60	5	n.d.^{a,A}	n.d.^{a,A}
		10	n.d.^{a,A}	n.d.^{a,A}
		15	n.d.^{a,A}	n.d.^{a,A}
		20	n.d.^{a,A}	n.d.^{a,A}
	65	5	n.d.^{a,A}	n.d.^{a,A}
		10	n.d.^{a,A}	n.d.^{a,A}
		15	n.d.^{a,A}	n.d.^{a,A}
		20	n.d.^{a,A}	n.d.^{a,A}
Pseudomonas aeruginosa	50	5	2.24±0.03^{a,A}	1.87±0.10^{b,A}
		10	2.13±0.04^{a,B}	1.66±0.09^{b,B}
		15	1.98±0.10^{a,CD}	1.46±0.04^{b,C}
		20	1.92±0.06^{a,C}	1.33±0.08^{b,D}
	55	5	1.83±0.04^{a,D}	n.d.^{b,E}
		10	1.50±0.05^{a,E}	n.d.^{b,E}
		15	n.d.^{a,F}	n.d.^{a,E}
		20	n.d.^{a,F}	n.d.^{a,E}
	60	5	n.d.^{a,F}	n.d.^{a,E}
		10	n.d.^{a,F}	n.d.^{a,E}
		15	n.d.^{a,F}	n.d.^{a,E}
		20	n.d.^{a,F}	n.d.^{a,E}
	65	5	n.d.^{a,F}	n.d.^{a,E}
		10	n.d.^{a,F}	n.d.^{a,E}
		15	n.d.^{a,F}	n.d.^{a,E}
		20	n.d.^{a,F}	n.d.^{a,E}
Essential oil+ *Pseudomonas aeruginosa*	50	5	2.17±0.06^{a,A}	1.86±0.09^{b,A}
		10	2.06±0.02^{a,B}	1.72±0.06^{b,AB}
		15	1.93±0.05^{a,C}	1.67±0.02^{b,B}
		20	1.73±0.04^{a,D}	1.55±0.06^{b,C}
	55	5	1.49±0.07^{a,E}	1.45±0.08^{a,C}
		10	1.15±0.07^{a,F}	n.d.^{b,D}
		15	n.d.^{a,G}	n.d.^{a,D}
		20	n.d.^{a,G}	n.d.^{a,D}
	60	5	n.d.^{a,G}	n.d.^{a,D}
		10	n.d.^{a,G}	n.d.^{a,D}
		15	n.d.^{a,G}	n.d.^{a,D}
		20	n.d.^{a,G}	n.d.^{a,D}
	65	5	n.d.^{a,G}	n.d.^{a,D}
		10	n.d.^{a,G}	n.d.^{a,D}
		15	n.d.^{a,G}	n.d.^{a,D}
		20	n.d.^{a,G}	n.d.^{a,D}

Values are presented as mean ± standard deviation (SD) of of 3 deer meat samples. $^{a-b}$ Differents superscripts lowercase letters indicate statistical different value within row (Duncan's MRT, $p \leq 0.05$). $^{A-G}$ Differents superscripts uppercase letters indicate statistical different value within column for each treatment (Duncan's MRT, $p \leq 0.05$). n.d.= not detected (value = 0.00).

Table S3. *Pseudomonas aeruginosa* count (log CFU/g) of sous-vide deer meat samples after 1 and 7 days of storage, treated in a water bath at temperatures between 50 and 65 °C for 5 to 20 min. Control: deer meat samples placed in polyethylene bags without vacuum. Control vacuum: deer meat samples vacuum-packed in polyethylene bags. Essential oil: deer meat samples treated with 1% EO and vacuum-packed. *Pseudomonas aeruginosa*: deer meat samples inoculated with *Pseudomonas aeruginosa* and vacuum-packed. Essential oil + *Pseudomonas aeruginosa:* deer meat samples treated with 1% EO and inoculated with *Pseudomonas aeruginosa* and vacuum-packed.

Treatment	Temperature (°C)	Time (min)	***Pseudomonas aeruginosa* (log CFU/g)**	
			**Days**	
			**1**	**7**
Control	50	5	n.d.[a,A]	n.d.[a,A]
		10	n.d.[a,A]	n.d.[a,A]
		15	n.d.[a,A]	n.d.[a,A]
		20	n.d.[a,A]	n.d.[a,A]
	55	5	n.d.[a,A]	n.d.[a,A]
		10	n.d.[a,A]	n.d.[a,A]
		15	n.d.[a,A]	n.d.[a,A]
		20	n.d.[a,A]	n.d.[a,A]
	60	5	n.d.[a,A]	n.d.[a,A]
		10	n.d.[a,A]	n.d.[a,A]
		15	n.d.[a,A]	n.d.[a,A]
		20	n.d.[a,A]	n.d.[a,A]
	65	5	n.d.[a,A]	n.d.[a,A]
		10	n.d.[a,A]	n.d.[a,A]
		15	n.d.[a,A]	n.d.[a,A]
		20	n.d.[a,A]	n.d.[a,A]
Control vacuum	50	5	n.d.[a,A]	n.d.[a,A]
		10	n.d.[a,A]	n.d.[a,A]
		15	n.d.[a,A]	n.d.[a,A]
		20	n.d.[a,A]	n.d.[a,A]
	55	5	n.d.[a,A]	n.d.[a,A]
		10	n.d.[a,A]	n.d.[a,A]
		15	n.d.[a,A]	n.d.[a,A]
		20	n.d.[a,A]	n.d.[a,A]
	60	5	n.d.[a,A]	n.d.[a,A]
		10	n.d.[a,A]	n.d.[a,A]
		15	n.d.[a,A]	n.d.[a,A]
		20	n.d.[a,A]	n.d.[a,A]
	65	5	n.d.[a,A]	n.d.[a,A]
		10	n.d.[a,A]	n.d.[a,A]
		15	n.d.[a,A]	n.d.[a,A]
		20	n.d.[a,A]	n.d.[a,A]
Essential oil	50	5	n.d.[a,A]	n.d.[a,A]
		10	n.d.[a,A]	n.d.[a,A]
		15	n.d.[a,A]	n.d.[a,A]
		20	n.d.[a,A]	n.d.[a,A]
	55	5	n.d.[a,A]	n.d.[a,A]
		10	n.d.[a,A]	n.d.[a,A]

		15	n.d.^{a,A}	n.d.^{a,A}
		20	n.d.^{a,A}	n.d.^{a,A}
	60	5	n.d.^{a,A}	n.d.^{a,A}
		10	n.d.^{a,A}	n.d.^{a,A}
		15	n.d.^{a,A}	n.d.^{a,A}
		20	n.d.^{a,A}	n.d.^{a,A}
	65	5	n.d.^{a,A}	n.d.^{a,A}
		10	n.d.^{a,A}	n.d.^{a,A}
		15	n.d.^{a,A}	n.d.^{a,A}
		20	n.d.^{a,A}	n.d.^{a,A}
Pseudomonas aeruginosa	50	5	2.20±0.03^{a,A}	1.90±0.07^{b,A}
		10	2.07±0.08^{a,B}	1.80±0.04^{b,A}
		15	1.93±0.04^{a,C}	1.58±0.06^{b,B}
		20	1.84±0.04^{a,D}	1.51±0.06^{b,B}
	55	5	1.76±0.02^{a,E}	1.39±0.03^{b,C}
		10	1.60±0.05^{a,F}	n.d.^{b,D}
		15	n.d.^{a,G}	n.d.^{a,D}
		20	n.d.^{a,G}	n.d.^{a,D}
	60	5	n.d.^{a,G}	n.d.^{a,D}
		10	n.d.^{a,G}	n.d.^{a,D}
		15	n.d.^{a,G}	n.d.^{a,D}
		20	n.d.^{a,G}	n.d.^{a,D}
	65	5	n.d.^{a,G}	n.d.^{a,D}
		10	n.d.^{a,G}	n.d.^{a,D}
		15	n.d.^{a,G}	n.d.^{a,D}
		20	n.d.^{a,G}	n.d.^{a,D}
Essential oil+ *Pseudomonas aeruginosa*	50	5	2.05±0.04^{a,A}	1.99±0.06^{a,A}
		10	1.93±0.06^{a,B}	1.88±0.09^{a,A}
		15	1.82±0.03^{a,C}	1.64±0.05^{b,B}
		20	1.71±0.05^{a,D}	1.50±0.05^{b,C}
	55	5	1.52±0.05^{a,E}	1.35±0.09^{b,D}
		10	1.32±0.05^{a,F}	n.d.^{b,E}
		15	n.d.^{a,G}	n.d.^{a,E}
		20	n.d.^{a,G}	n.d.^{a,E}
	60	5	n.d.^{a,G}	n.d.^{a,E}
		10	n.d.^{a,G}	n.d.^{a,E}
		15	n.d.^{a,G}	n.d.^{a,E}
		20	n.d.^{a,G}	n.d.^{a,E}
	65	5	n.d.^{a,G}	n.d.^{a,E}
		10	n.d.^{a,G}	n.d.^{a,E}
		15	n.d.^{a,G}	n.d.^{a,E}
		20	n.d.^{a,G}	n.d.^{a,E}

Values are presented as mean ± standard deviation (SD) of of 3 deer meat samples. $^{a-b}$ Differents superscripts lowercase letters indicate statistical different value within row (Duncan's MRT, $p \leq 0.05$). $^{A-G}$ Differents superscripts uppercase letters indicate statistical different value within column for each treatment (Duncan's MRT, $p \leq 0.05$). n.d.= not detected (value = 0.00).
